# How Do People Attribute Income-Related Inequalities in Health? A Cross-Sectional Study in Ontario, Canada

**DOI:** 10.1371/journal.pone.0085286

**Published:** 2014-01-13

**Authors:** Aisha Lofters, Morgan Slater, Maritt Kirst, Ketan Shankardass, Carlos Quiñonez

**Affiliations:** 1 Centre for Research on Inner City Health, St. Michael's Hospital, Toronto, Ontario, Canada; 2 St. Michael's Department of Family & Community Medicine, University of Toronto, Toronto, Ontario, Canada; 3 Dalla Lana School of Public Health, University of Toronto, Toronto, Ontario, Canada; 4 Department of Psychology, Wilfrid Laurier University, Waterloo, Ontario, Canada; 5 Faculty of Dentistry, University of Toronto, Toronto, Ontario, Canada; Aga Khan University, Pakistan

## Abstract

**Context:**

Substantive equity-focused policy changes in Ontario, Canada have yet to be realized and may be limited by a lack of widespread public support. An understanding of how the public attributes inequalities can be informative for developing widespread support. Therefore, the objectives of this study were to examine how Ontarians attribute income-related health inequalities.

**Methods:**

We conducted a telephone survey of 2,006 Ontarians using random digit dialing. The survey included thirteen questions relevant to the theme of attributions of income-related health inequalities, with each statement linked to a known social determinant of health. The statements were further categorized depending on whether the statement was framed around blaming the poor for health inequalities, the plight of the poor as a cause of health inequalities, or the privilege of the rich as a cause of health inequalities.

**Results:**

There was high agreement for statements that attributed inequalities to differences between the rich and the poor in terms of employment, social status, income and food security, and conversely, the least agreement for statements that attributed inequalities to differences in terms of early childhood development, social exclusion, the social gradient and personal health practices and coping skills. Mean agreement was lower for the two statements that suggested blame for income-related health inequalities lies with the poor (43.1%) than for the three statements that attributed inequalities to the plight of the poor (58.3%) or the eight statements that attributed inequalities to the privilege of the rich (58.7%).

**Discussion:**

A majority of this sample of Ontarians were willing to attribute inequalities to the social determinants of health, and were willing to accept messages that framed inequalities around the privilege of the rich or the plight of the poor. These findings will inform education campaigns, campaigns aimed at increasing public support for equity-focused public policy, and knowledge translation strategies.

## Introduction

Income-related health inequalities in Canada are well recognized by the health, policy, and research communities, as is the role of the social determinants of health (SDOH), such as education, housing, and job security, in producing and maintaining these inequalities [1]–[9]. Indeed, it has been argued that income inequality is the key determinant of health among Canadians [10]. These stakeholders also recognize that society bears much of the responsibility for health inequality, and accordingly, that governmental policies and programs can mitigate marginalization [11], [12]. As a result, the World Health Organization (WHO) Commission on the Social Determinants of Health endorsed the incorporation of the SDOH into governmental policies and programs [13]. In Canada, there has also been a move towards addressing health inequalities at the local, provincial, and national levels, including the promotion of the SDOH by select local public health units in the province of Ontario (e.g. Sudbury and District Health Unit and Peterborough County-City Health Unit), the use of health equity impact assessment tools to improve decision-making in Ontario [14] and cross-sectoral policies in the province of Quebec [15], and the establishment of equity-focused research priorities within federal research funding bodies such as the Canadian Institutes of Health Research [16]. Despite these initial steps, substantive policy change has yet to be realized in most Canadian jurisdictions, including Ontario, the country's most populous and diverse province. This relative inaction may be due to competing social and political interests, but may also reflect a lack of widespread public attention to the need for such policies [17]. Tellingly, the WHO Commission endorsed raising public awareness regarding the SDOH as a key step to “closing the gap” in health “within a generation”, suggesting that public awareness may serve as a significant motivating factor for policymakers if it existed [13].

Our previous research suggests that only a small majority of Ontarians are aware of income-related health inequalities in the province, with 53% agreeing that ‘the rich are much healthier than the poor’ [18]. As well, we have shown that certain sociodemographic characteristics, such as age, education, and political affiliation, are associated with awareness of income-related health inequalities [18]. These and other characteristics might also influence how one attributes the causes of health inequalities. Attribution theory posits that individuals understand social phenomena by attributing the causes of such phenomena to internal or external factors (i.e. caused by individual dispositions or by social factors outside of the individual's control) [19]. This theory also suggests that if someone has not experienced a particular situation or condition, such as those that make up the SDOH, then he or she is less likely to recognize the role that the SDOH may play in producing and maintaining inequalities. He or she may then subsequently see no reason to support equity-focussed policies [19], [20]. This tendency of the observer to then attribute outcomes to internal factors instead of to external factors has been called “the fundamental attribution error” [21]. In this way, the public's beliefs and judgments about the SDOH, and their potential support for equity-related policies, may be influenced by their sociodemographic circumstances [19], [22]–[24].

An understanding of how Ontarians attribute income-related health inequalities can be used to inform effective framing of messages aimed at increasing public awareness of inequalities and support for policy change to promote health equity. Effective messages should include emphasis on the importance of external factors, but we must also ascertain what messages the general public, and population subgroups, are willing to accept. For example, Niederdeppe et al.'s Message Design Strategy Framework for Raising Awareness of SDOH and Population Health Disparities states that messaging can frame inequalities as being within the control of the disadvantaged individual, being beyond the control of that individual, or some combination of the two, with the most effective messaging emphasizing external factors but acknowledging the role of the individual[19]. Although this framework was developed in the American context, it follows that how the message on health inequalities is framed can affect how willing Ontarians will be to absorb the messages. Linked to this is the push in the health promotion literature to move beyond understanding the plight of disadvantaged and poor communities toward an appreciation of the maintenance of privilege by advantaged groups [25], [26]. As a result, messages around income-related health inequalities could be more or less agreeable to the Ontario public, and to various subgroups, depending on if the messages frame the poor as being responsible for their relatively unequal and worse health (what we refer to as ‘blame’), or if they explain inequalities as related to external factors in broader society such as the social advantages or privilege of the rich, or the social disadvantages of the poor (what we refer to as ‘privilege’ and ‘plight’ respectively).

With these considerations in mind, this paper reports on survey results of how Ontarians attribute differences in health between the rich and poor. The data presented here are part of a larger body of work where the overarching aim is to better understand how to increase public support for health equity by exploring Ontarians' awareness of income-related health inequalities, their attributions of these inequalities, and their opinions about solutions to inequalities. Specifically, this paper examines Ontarians' attributions of income-related health inequalities relative to the SDOH, their attributions relative to the framing of messages, and how various sociodemographic factors influence these attributions.

## Methods

### Ethics Statement

The study, and its consent procedures, received approval by the University of Toronto's Office of Research Ethics. Individuals were asked if they would participate in the survey, and when they verbally agreed, this was taken to imply consent. This is common practice in telephone interview surveys that are deemed to present little or no risk to participants, so no written consent is generally obtained. As well, it was not feasible to obtain written consent due to the nature of the survey (i.e. conducted by telephone by a third party market-based research firm via random digit dialling). All telephone surveys were recorded for quality control purposes, and all responses were entered real-time using computer-assisted telephone interview technology. The process, as documented by the market-based research firm, indicated that of 69,906 numbers called, there were a total of 33,530 individuals asked to participate with 9.24% of persons asked to complete the survey doing so.

### Study Sample

Details of the study methods have been previously published [18]. Briefly, we surveyed 2,006 Ontarians aged 18 years and over through a telephone interview survey using random digit dialing. A sample size calculation indicated that this would provide a 3.0% margin of error with 95% confidence relative to the Ontario population.

### Survey

The survey included questions pertaining to three broad themes: (1) awareness of income-related health inequalities, (2) attributions of income-related health inequalities, and (3) possible solutions to income-related health inequalities. Results related to the first theme have been previously published [18]. The outcomes examined in this analysis relate to the second theme only.

We analyzed responses to thirteen Likert items, with each attribution statement linked to a particular SDOH (Table 1). As many typologies for the SDOH exist, each with benefits and limitations when considering income-related health inequalities, we have drawn our list of determinants from a variety of sources, including the Public Health Agency of Canada and the Chief Public Health Officer's Report on the State of Public Health in Canada to ensure relevance to the Canadian context [27]. The list of determinants was chosen by consensus by the research team, with the goal of achieving a thorough list and a survey that would not be onerous for respondents. The statements were further categorized depending on whether the statement was framed around blaming the poor for health inequalities (two statements), the plight of the poor as a cause of health inequalities (three statements), or the privilege of the rich as a cause of health inequalities (eight statements). Due to the focus on the SDOH, statements in the latter two categories predominated.

**Table 1:** Thirteen statements presented to survey respondents on attributions of income-related health inequalities, the social determinant of health to which the statement attributed inequalities, and whether the statement was framed around blaming the poor, the plight of the poor, or the privilege of the rich.

Statement	Social Determinant of Health	Message Framing: Blame, Plight, or Privilege
The poor are less healthy because of their lifestyles - they smoke and drink more, don't exercise and eat junk food	Health behaviours	Blames the poor
The poor spend what money they have unwisely because they do not want to feel excluded from the good things in life	Social exclusion	Blames the poor
The poor smoke and drink more to help them cope with the stress and anxiety in their lives; that is why they have poor health	Personal health practices and coping skills	Plight of the poor
The poor are less healthy because they have more stress and anxiety in their lives than those who are better off	Stress	Plight of the poor
If you work in a poorly paying job, the insecurity you feel can have a bad effect on your health	Employment and working conditions	Plight of the poor
The rich are healthier because they live in better houses in better neighbourhoods	Environment and housing	Privilege of the rich
The rich are healthier because they have money to buy things that make them healthy	Income	Privilege of the rich
Even though everyone in Ontario has access to medical care, the rich get more out of the health care system than the poor	Access to health care	Privilege of the rich
The rich have more choices and more control over their lives and health than the poor	Social status	Privilege of the rich
The rich are healthier because they have better access to high quality food	Food security	Privilege of the rich
Some people are at the top of the social ladder and some people are at the bottom; this is why the rich are healthier than the poor	Social gradient	Privilege of the rich
The rich are healthier because their childhood experiences are much better	Early childhood development	Privilege of the rich
The rich are healthier because they have more education and know how to stay healthy	Education and literacy	Privilege of the rich

For all statements, participants were given the options of strongly agreeing, agreeing, disagreeing, strongly disagreeing, providing a neutral response, or refusing to answer. The proportion refusing to answer ranged from 0.2% to 2.8%. To investigate participant characteristics that may influence how one attributes health inequalities, political affiliation and demographic information were also collected. Demographic characteristics used in this analysis include sex, age group (18–34 years, 35–54 years, 55+ years), area of residence (urban vs. rural), immigration status (immigrated more than 10 years ago, immigrated 10 years ago or less, Canadian-born), visible minority status, total annual household income (< $20,000, $20,000–< $40,000, $40,000 – <$60,000, $60,000 – <$80,000, $80,000 – <$100,000, $100,000+), highest attained education (high school diploma or lower versus higher than high school), and whether participants were employed at the time of the survey. Political affiliation was gauged in response to the question, “If the election were being held today, do you think you would vote for the Progressive Conservative, Liberal, New Democratic Party (NDP), Green, or some other candidate?” The former three parties are the parties currently represented in the Ontario legislative assembly, and can very generally be defined as right to left wing, respectively. Participants who indicated affiliation with the Green Party or another candidate were classified as ‘Other’. We also examined a fifth category of political affiliation that comprised participants who either didn’t know who they would vote for or who refused to answer the question.

### Analysis

We tabulated the proportion of survey responses to each of the thirteen statements. We then classified responses as a binary measure indicating some level of agreement, versus some level of disagreement or a neutral response. To determine the association of sociodemographic characteristics with survey responses, we conducted multivariable logistic regression. For this analysis, all characteristics that were associated with agreement, based on a significance level of 0.2, were eligible for inclusion in the model. Spearman correlation coefficients between all significant subgroup characteristics were examined to identify collinearity between predictors. We then used a manual backward stepwise approach to identify a parsimonious list of characteristics that independently predicted agreement with the attribution statements.

For all analyses, data were weighted to provide estimates that were representative of the provincial population. Weights were based on provincial age and sex distributions according to the 2006 Canadian Census. SAS Version 9.3 was used for all analyses (SAS Institute, Cary, NC).

## Results

The participation rate and demographic profile of study participants are described elsewhere [18]. Study participants were generally representative of the Ontario population based on the 2006 Census. Briefly, females composed 52% of the sample. Close to 30% of participants reported an annual household income of $40,000 or less, 26.5% reported a high school diploma or less as the highest attained education, and 6.2% reported current unemployment. When asked who they would vote for if an election were being held today, 24.5% reported Progressive Conservative, 21.8% reported Liberal, 11% reported NDP, 13.2% opted for another party, and 29.6% of participants did not know who they would vote for or refused to answer the question.

**Figure 1:**
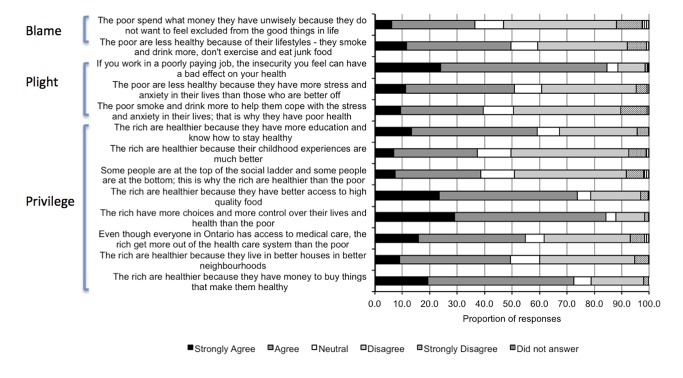
Distribution of responses.

Almost 85% strongly agreed or agreed that income-related health inequalities could be attributed to the poor having greater job insecurity, or to the rich having more choices and control over their lives than the poor (Figure 1). There was also high agreement for statements that implicated differences in food security (74%) and in income that enabled the rich to “buy things that make them healthy” (72%). Conversely, the least agreement was seen for statements that attributed income-related health inequalities to unhealthy coping behaviours (40%), the social gradient (39%), unwise spending amongst the poor (37%), and better early childhood experiences among the rich (37%). The framing of the message seemed to affect likelihood of agreement. Mean agreement was lower for the two statements that suggested blame for income-related health inequalities lies with the poor (43.1%) than for the three statements that attributed inequalities to the plight of the poor (58.3%) or the eight statements that attributed inequalities to the privilege of the rich (58.7%).

Tables 2 and 3 describe the results of multivariable logistic regression, which identified sociodemographic characteristics associated with agreement with statements. Of the characteristics that met inclusion for the multivariate model based on univariate analyses, only two, immigration status and visible minority, appeared to be correlated (r = 0.4982). We included visible minority over immigration status in multivariate models, as this is arguably a better representation of how one may be viewed socially [28]. Older age was associated with a higher likelihood of agreement with almost all statements, but with only one of the three “plight” statements. Men were more likely than women to agree with both “blame” statements and less likely to agree that “the rich get more out of the health care system than the poor”. Visible minority status, lower income, lower education, and certain political affiliations were significantly associated with agreement across statements. Visible minorities were more likely to agree with both “blame” statements, with most (two of the three) “plight” statements, and with statements relevant to the social gradient and childhood experiences. Respondents tended to be more likely to agree across statements as their income decreased, with the difference in response most striking for “the poor are less healthy because they have more stress and anxiety in their lives than those who are better off”. People of low education attainment tended to be more likely to agree across statements but were less likely to agree that “the rich are healthier because they have more education and know how to stay healthy”. Supporters of the NDP, Canada's most left-leaning party, were less likely to agree that the poor are less healthy because of their lifestyles, were more likely to agree that the rich are healthier because of access to high quality food, and along with “Other” voters (many of whom supported the Green party, which is generally known for an environmental emphasis yet fiscal conservatism), were more likely to agree that the rich get more out of the health care system than the poor. Supporters of the Progressive Conservatives (PCs), Canada's most right-leaning party, were more likely to agree that “the poor spend what money they have unwisely because they do not want to feel excluded from the good things in life”.

**Table 2:** Results of multivariate logistic regression: statements framed around blaming the poor or the plight of the poor.

	Blame		Plight		
	Health behaviours	Social exclusion	Personal health practices and coping skills	Stress	Employment and working conditions
Proportion agreement	49.6	36.5	39.5	50.9	84.6
Age group [Reference: 18–34]					
35–54	1.27 (0.99 – 1.62)	1.02 (0.79 – 1.34)	1.38 (1.07 – 1.79)		
55+	2.70 (2.05 – 3.55)	1.41 (1.06 – 1.88)	1.79 (1.36 – 2.36)		
Sex (Male)	1.61 (1.32 – 1.98)	1.80 (1.45 – 2.23)	1.38 (1.12 – 1.69)		
Residence in a Census Metropolitan Area					
Place of birth and immigration status [Reference: Born in Canada]					
Born outside of Canada and immigrated >10y ago					
Born outside of Canada and immigrated < = 10y ago					
Visible minority	1.46 (1.11 – 1.92)	1.90 (1.43 – 2.53)	1.75 (1.33 – 2.30)	1.43 (1.10 – 1.87)	
Annual household income [Reference: <$20,000]					
$20,000 – <$40,000		0.96 (0.65 – 1.42)	0.77 (0.53 – 1.13)	0.79 (0.54 – 1.17)	
$40,000 – <$60,000		0.83 (0.57 – 1.23)	0.73 (0.50 – 1.07)	0.71 (0.48 – 1.04)	
$60,000 – <$80,000		0.69 (0.46 – 1.03)	0.56 (0.38 – 0.83)	0.51 (0.35 – 0.76)	
$80,000 – <$100,000		0.58 (0.38 – 0.90)	0.66 (0.44 – 1.00)	0.47 (0.31 – 0.72)	
> = $100,000		0.50 (0.34 – 0.74)	0.43 (0.29 – 0.62)	0.39 (0.27 – 0.57)	
Highest education < = highschool diploma		1.73 (1.36 – 2.22)	1.39 (1.10 – 1.77)	1.44 (1.13 – 1.83)	
Political affiliation [Reference: Don't know/refused]			-		
PC	1.00 (0.76 – 1.33)	1.34 (1.00 – 1.79)			
Liberal	1.23 (0.92 – 1.64)	0.81 (0.59 – 1.10)			
NDP	0.66 (0.46 – 0.94)	1.06 (0.73 – 1.54)			
Other	1.04 (0.74 – 1.46)	1.01 (0.71 – 1.44)			

The variable for ‘currently unemployed’ not shown as it is not included in any final model.

**Table 3:** Results of multivariate logistic regression: questions framed around the privilege of the rich.

	Income	Environment and housing	Access to health care	Social status	Food security	Social gradient	Early childhood development	Education and literacy
Proportion agreement	72.4	49.4	54.9	84.2	73.8	38.6	37.4	59.1
Age group [Reference: 18–34]								
35–54	1.27 (0.98 – 1.65)	1.34 (1.02 – 1.64)		1.65 (1.21 – 2.25)	1.29 (0.99 – 1.68)	1.64 (1.26 – 2.13)	1.49 (1.15 – 1.93)	1.64 (1.30 – 2.08)
55+	1.46 (1.10 – 1.93)	1.61 (1.24 – 2.09)		2.75 (1.89 – 4.03)	1.64 (1.22 – 2.19)	2.23 (1.68 – 2.95)	2.35 (1.77 – 3.11)	2.52 (1.93 – 3.28)
Sex (Male)			0.72 (0.59 – 0.88)			1.41 (1.15 – 1.74)		
Residence in a Census Metropolitan Area							1.41 (1.11 – 1.79)	1.49 (1.20 – 1.86)
Place of birth and immigration status [Reference: Born in Canada]								
Born outside of Canada and immigrated >10y ago		1.62 (1.26 – 2.08)						
Born outside of Canada and immigrated < = 10y ago		1.54 (0.97 – 2.44)						
Visible minority						1.70 (1.29 – 2.24)	2.40 (1.82 – 3.15)	
Annual household income [Reference: <$20,000]	-							
$20,000 – <$40,000		0.76 (0.53 – 1.10)	0.60 (0.41 – 0.88)			0.73 (0.52 – 1.12)		
$40,000 – <$60,000		0.66 (0.46 – 0.96)	0.64 (0.43 – 0.62)			0.77 (0.52 – 1.12)		
$60,000 – <$80,000		0.58 (0.39 – 0.84)	0.53 (0.36 – 0.79)			0.73 (0.49 – 1.09)		
$80,000 – <$100,000		0.48 (0.32 – 0.72)	0.46 (0.30 – 0.69)			0.54 (0.36 – 0.83)		
> = $100,000		0.45 (0.31 – 0.64)	0.61 (0.42 – 0.88)			0.54 (0.37 – 0.80)		
Highest education < = highschool diploma						1.39 (1.09 – 1.77)		0.71 (0.56 – 0.89)
Political affiliation [Reference: Don't know/refused]								
PC			1.05 (0.80 – 1.38)		0.77 (0.57 – 1.04)			
Liberal			1.08 (0.81 – 1.42)		1.00 (0.73 – 1.38)			
NDP			1.50 (1.05 – 2.12)		1.47 (0.97 – 2.24)			
Other			1.76 (1.25 – 2.46)		1.05 (0.72 – 1.54)			

The variable for ‘currently unemployed’ not shown as it is not included in any final model.

## Discussion

In this survey of Ontarians, we found that respondents were most willing to attribute income-related health inequalities to differences between the rich and poor in terms of employment, social status, income and food security, and least willing to attribute inequalities to differences in terms of early childhood development, social exclusion, the social gradient and personal health practices and coping skills. These distinctions may reflect a better understanding of some social determinants by the public than others, and are important for health equity advocates to consider when working to create widespread support for health equity-focused public policy in Ontario. These findings identify policy solutions that the general public may be more willing to support at present, as well as issues where more education is needed to improve popular understanding and support. We also found that respondents were generally more likely to agree with statements that were framed around the plight of the poor or the privilege of the rich and less likely to agree with statements that implied the poor were to blame for income-related health inequalities. This finding suggests a willingness on the part of Ontarians to accept a more macro-social understanding of the determinants of health. While there was no noticeable preference for the “plight” frame over the “privilege” frame, it must be noted that mean agreement for neither exceeded 59%, signifying room for improvement in the public's understanding of social responsibility.

Attribution theory suggests that certain SDOH may have resonated more with certain respondents because of the role of lived experience [19], [20]. In our analysis, older respondents, visible minorities, and people of lower income were generally more likely to attribute inequalities to the SDOH. Ruetter et al. have argued that these characteristics influence and reflect social standing, suggesting that differences in responses for these subgroups reflect their lived experience with the SDOH and with health inequalities [29]. For example, we found that visible minorities were more likely to understand the importance of social exclusion as a SDOH for low-income Ontarians, which may reflect their personal experience with racism, discrimination and social exclusion [30], [31]. Similarly, women were more likely to agree that “even though everyone in Ontario has access to medical care, the rich get more out of the health care system than the poor”, which, again, may reflect their experience with discrimination in the health care system and with the inappropriateness of some services [32], [33].

Attributions are influenced not just by personal experience, but also by one's socialization (i.e. the norms and values to which a person conforms to because of the groups with which they identify) [34]. In this study, political affiliation was associated with how respondents attributed inequalities, with left-leaning respondents being less willing to agree with the statement that attributed inequalities to individual lifestyle choices and more willing to agree with statements that criticized existing social structures around access to health care and food insecurity. Similarly, a more conservative socialization and ideology may equate to less willingness to attribute inequalities to the SDOH and to contextual factors [35]. Nevertheless, the reasons for the other sociodemographic differences that we observed are not clear and need to be explored, but may again relate to socialization. For example, men and visible minorities were more likely to agree with statements that blamed the poor for income-related health inequalities, and statements that were framed around the plight of the poor resonated less with older respondents. Whether based on a lack of lived experience or social norms, we have highlighted certain groups that may be harder to convince about the importance of the SDOH and accordingly of policies that reflect an understanding of these determinants.

We found a number of other Canadian studies that corroborate several of our findings. For example, Ruetter et al. have explored attributions of poverty and health, and similarly have shown that Canadians generally have a good understanding of the effects of poverty on health and quality of life [36], [37]. Canadians living in the province of Alberta were most likely to explain the relationship between poverty and poor health with drift (whereby people become poor after they get sick) or structural (whereby poor people are unhealthy because of living circumstances) hypotheses than with myth (whereby there is no link between poverty and health) or behavioural (where poor people exhibit behaviours that make them unhealthy) hypotheses [37]. Conservatism was the most consistent predictor of non-adherence to the structural hypothesis in one study [37]. Reutter et al. (2006) also conducted a telephone interview in two large Canadian cities on how the public attributes poverty [29]. Participants were generally most likely to attribute poverty to structural causes and least likely to attribute it to individual causes, and most knew that there was a relationship between poverty and health. Demographic variables explained a modest amount of variance in possible reasons for poverty. Men and people with less education were most likely to attribute poverty to laziness, suggesting less of an understanding of social determinants in general. In contrast, in the American literature, attributions of poverty that focus on the poor individual instead of on the failings of social structures tend to dominate [19], [23], [38], [39], which may reflect a more individualistic ethos or a more right-leaning political landscape in the U.S. [40]. Other relevant literature corroborates our finding that perceptions of the SDOH vary on the basis of political affiliation, with conservatives being more likely than liberals to disagree with the impact of the SDOH [41]–[43].

Our findings will be informative for the process of devising effective knowledge translation activities and messaging. We have shown that this sample of Ontarians was generally willing to accept messages framed around the plight of the poor or the privilege of the rich, with no strong preference for either. Messages that lay responsibility on the poor for inequalities seem likely to be less appealing to the public. Our results also suggest that, to be most effective, messages may need to be framed differently for different sociodemographic groups, such as older Ontarians, men or visible minorities [38], [43]. Essentially, knowledge translation and communication experts will have to work to counter both the “fundamental attribution error” and certain imposed norms. Although perceptions exist that the SDOH are not newsworthy and that the mainstream media are unlikely to provide coverage on the SDOH compared to other health determinants, the media have the potential to be an important partner in dissemination ventures [19], [29], [39], [44]. Media messaging can provide the public with exposure to health inequalities and can influence opinions about which SDOH-based policies should be implemented. Therefore, these perceptions of the media will have to be challenged by health equity advocates moving forward.

This study has several limitations. First, we used telephone sampling to obtain respondents. Telephone surveys exclude those without conventional landlines and accordingly, might under-sample people of lower socioeconomic position [18]. Future surveys should include cellular telephone users in order to maintain relevance. However, our sample had similar annual household income to the Ontario population as per the 2006 Census (29.8% with an annual household income of $40,000 or less versus 24.0% with an annual household income of less than $40,000) [18], [45]. As well, to combat any deviations from having a representative sample, our survey data were statistically weighted to be representative of Ontario adults in terms of composition by age and sex. Second, our response rate was low. However, this is typical of the response for random digit dialing surveys. To ensure representativeness, we constructed a quota sample whereby quotas for sex, age and regional representation were filled. Third, social desirability bias may have prevented respondents from verbally agreeing with statements that seemed to lay blame, even if they did in reality hold that viewpoint. Fourth, our constructs of blame, plight and privilege were not tested for their validity. However, their use has been supported by the health promotion and health inequities literature [19], [25], [26]. Finally, our chosen list of social determinants of health was not exhaustive. Using a broader set of determinants may have been more informative for policy makers. However, we wanted to limit the burden on survey respondents.

## Conclusions

Although a majority of this sample of Ontarians were aware of income-related health inequalities [18], attributed inequalities to the SDOH, and were willing to accept messages that framed inequalities around the privilege of the rich or the plight of the poor, our results indicate that there is still significant room for improvement in their understanding of the root causes of inequalities. These findings will inform education campaigns, campaigns aimed at increasing public support for equity-focused public policy, and knowledge translation strategies. Our future work will focus on perceived solutions to income-related health inequalities among this sample of Ontarians, which will provide a more complete picture of beliefs and attitudes toward income-related health inequalities, and help to guide policymakers as they work toward building political will for equity-related public policy in the province.
